# Dairy sheep and goats prefer the single components over the mixed ration

**DOI:** 10.3389/fvets.2022.1017669

**Published:** 2022-10-14

**Authors:** Roxanne Berthel, Michael Simmler, Frigga Dohme-Meier, Nina Keil

**Affiliations:** ^1^Centre for Proper Housing of Ruminants and Pigs, Agroscope Tänikon, Veterinary Affairs and Food Safety Office, Ettenhausen, Switzerland; ^2^Digital Production, Agroscope Tänikon, Ettenhausen, Switzerland; ^3^Ruminant Research Group, Agroscope Posieux, Posieux, Switzerland

**Keywords:** feed preference, ruminant, sheep, goat, silage, feed choice

## Abstract

Mixed rations provide ruminants with a balanced diet by aiming to prevent selective feeding. However, this is a natural behavior of sheep and goats based on their dietary needs and the nutritional properties of feedstuffs. Therefore, the present study investigates non-lactating dairy sheep's and goats' acceptance of a mixed ration when it is offered as choice next to its single components. Because all offered feeds were of comparable nutritional value, the animals were expected to not show a particular preference. Twelve pairs of sheep and goats each, were offered three different feeds simultaneously for 5 replicate days. Two feeds consisted of a single component, hay (H) or grass-silage (G) of similar nutritional value. The third feed was a mixed ration (M) including both single-feed components in a 50:50 dry matter (DM) ratio. Feeds were offered *ad libitum* twice daily. The animals' intake of each feed was recorded at six time points per day by weighing the leftovers. Feed preference was expressed as the natural logarithm of the ratio of the intake of the single component to the intake of M and analyzed using linear mixed-effects models. Additionally, the animals' first choices after gaining access to the feeds were recorded at each weighing event and analyzed using an item response tree generalized mixed-effects model. The sheep's average daily DM intake was 59 (±11)% G, 26 (±10)% H, and 15 (±10)% M (mean ± standard deviation). Goats consumed an average of 56 (±13)% G, 37 (±12)% H, and 7 (±6)% M daily. Both species preferred the single components to M in all observation periods. The proportions of the three feeds consumed differed throughout the day and between species. For both species, the estimated probability that an animal chooses a single component over M first was over 94% at all time points. These results show that, contrary to our expectations, non-lactating dairy sheep and goats prefer single components over a mixed ration of the same components and similar nutritional value. This might be caused by the animals seeking to diversify their feed throughout the day independent of apparent nutritional values and/or because sensory properties of the single components, indicating palatability, are relevantly reduced by mixing.

## Introduction

The use of mixed rations played a major part in the intensification of beef and dairy cattle production in recent decades (1). Mixed rations are fed as partial mixed rations (PMR) or total mixed rations (TMR). In PMRs, usually, roughage feed components are mixed and other components, such as concentrates, are offered separately. TMRs, on the other hand, contain all ingredients of the diet, including minerals and concentrates. Feeding livestock mixed rations is labor-efficient, reduces feed refusal, and provides nutritional advantages for the animals (1). Mixing components allows to combine less palatable feedstuffs with more palatable ones into a balanced diet and to easily adapt to various production levels (1). Mixed rations also enable all animals in a herd to access the same feed by preventing individual animals from monopolizing access to preferred feedstuffs (2) such as concentrates (3). Additionally, mixed rations reduce sorting for feed components in cattle (1), resulting in more consistent feed quality over time. This increases feed intake, especially for animals that reach the feed later than others in the herd (4), thus increasing animal productivity and feed efficiency (5).

In small ruminants (sheep and goats), the use of mixed rations is not yet as widespread as in cattle. However, the general worldwide trend toward fewer farms with larger herd sizes suggests that this labor-efficient feeding system will also be increasingly used for small ruminants. The effects of feeding mixed rations on productivity in small ruminants have been investigated, but the results are less consistent than for cattle. Monzón-Gil et al. (6) demonstrated that TMR feeding increased feed intake and milk yield in goats compared to single component feeding. Görgülü et al. (7) found that goats freely choosing the ratio of feed components (of the compared TMR) showed higher dry matter intake and higher milk yield than TMR-fed goats, although milk production efficiency was better on the TMR diet. In contrast, Yurtseven et al. (8) found that in sheep TMR feeding had no effect on milk production performance compared to free-choice feeding with the feeds of the compared TMR.

To better understand the effects of mixed-ration feeding in small ruminants, it is necessary to consider these animals' distinct feeding behavior. The ancestors of sheep and goats evolved predominantly in harsh environments and thus developed very selective foraging and feeding behaviors as an adaptation to seasonal and local variations in the availability of feed plants (9). Domestic sheep and goats kept in natural and semi-natural environments use selective browsing to adapt their intake to their nutritional needs (9–11). Sheep and goats also sort components of a feed (12) and choose among different feeds in indoor feeding conditions according to the varying nutritional needs of their current physiological stage (12). Therefore, it is unclear whether mixed rations are appropriate for sheep and goats as these rations are explicitly designed to limit selective feeding (13).

Previous studies have found that sheep and goats select their feed based on nutritional aspects in order to obtain a diet that meets their nutritional requirements. For instance, sheep and goats have both shown a preference for forages with higher organic matter digestibility and lower fiber content, preferring, for example, leafy grass hay to mature grass hay or straw (14). In short-term preference tests (3 min sessions), goats' feed choices were more influenced by the type of starch than by forage-to-concentrate ratios; they preferred starches that degrade rapidly in the rumen to those that degrade slowly (15). In a three-week feeding experiment, sheep ate more feeds supplemented with NaHCO_3_ than unsupplemented feeds (16). Goats have also been shown to adapt their concentrate intake based on its crude protein concentration, eating less soybean-based concentrate (which is high in crude protein content) than chickpea-based concentrate (which is lower in crude protein), leading to a consistent percentage of crude protein intake in the total diet (17).

Additionally, small ruminant adapt their feed intake and choices based on what feeds they have already consumed. It is assumed that they do this by monitoring the current condition of the rumen (18). For example, sheep's consumption of low-energy-density feeds depends on the carbohydrate sources of other feeds consumed (16). Thus, although small ruminants prefer energy-dense feeds (19), they apparently substitute their diet with feedstuff higher in fiber contents if necessary to balance the ruminal pH (20). This might explain why free-choice-fed goats prefer different feeds at different times of day (7). When foraging in natural and semi-natural environments both species prefer different plant species when the available variety is not restricted (11). But for harvested feeds of restricted number of options [six forages (14)] and for artificially flavored feeds (21) sheep and goats show similar preferences.

Based on the studies described above, one would expect that sheep and goats will not show a preference for a particular feed if all offered feeds meet the animals' nutritional requirements and are comparable in terms of properties such as energy density and fiber content. Therefore, the aim of this study was to assess non-lactating dairy sheep's and goats' acceptance of a mixed ration when the single components of that ration, grass silage and hay, are offered at the same time. All three feeds (mixed, grass silage, and hay) had similar nutritional value and met the animals' nutritional needs. We therefore hypothesized that, on average, all three feeds would be consumed by both species in similar amounts regardless of the time of day.

## Materials and methods

### Animals and housing conditions

The experiments were conducted in October 2020 at the Agroscope Research Station in Ettenhausen, Switzerland. The sample included 24 female dairy goats (10 Saanen, 11 Chamois Colored goats, 3 crossbreeds) and 24 female dairy sheep (20 Lacaunne 4 East Friesian sheep). All animals were 3 years old and had never been lactating or pregnant. At the start of the experiment, the mean body weight of the goats was 67.5 (standard deviation ±6.9) kg, and the mean body weight of the sheep was 78.4 (±7.9) kg. During the experimental phase, the goats and sheep gained an average of 1.94 and 1.19 kg, respectively.

Prior to the experimental phase, the sheep and goats were kept in the same stable in an outdoor climate with one pen for each species. The goat pen had a total area of 53 m^2^ (13.6 × 3.9 m), including a straw-bedded deep litter area of 40 m^2^ and an elevated feeding area, 0.95 m wide, along the long axis of the pen. The deep litter area was equipped with three benches (2.4 × 0.62 m; height: 0.6 m) and three round tables (diameter: 1.1 m; height: 0.8 m). The sheep pen had a total area of 42 m^2^ (11.7 × 3.6 m) with a deep litter area of 33 m^2^ and an elevated feeding area, 0.8 m wide, along the long axis of the pen. Each pen had three drinkers for *ad libitum* access to water and one mineral supply. Feed troughs with a palisade feeding fence (35 and 40 cm feeding space per animal for goats and sheep, respectively) were placed along the entire long axis of each pen.

All animal care and experimental procedures were performed in accordance with the relevant legislative and regulatory requirements and the ASAB/ABS Guidelines for the Use of Animals in Research (22). The Cantonal Veterinary Office, Thurgau, Switzerland (Approval No. TG10/18–30902) approved all procedures involving animal handling and treatment.

### Experimental setup and procedures

#### Experimental pens

**Figure 1:**
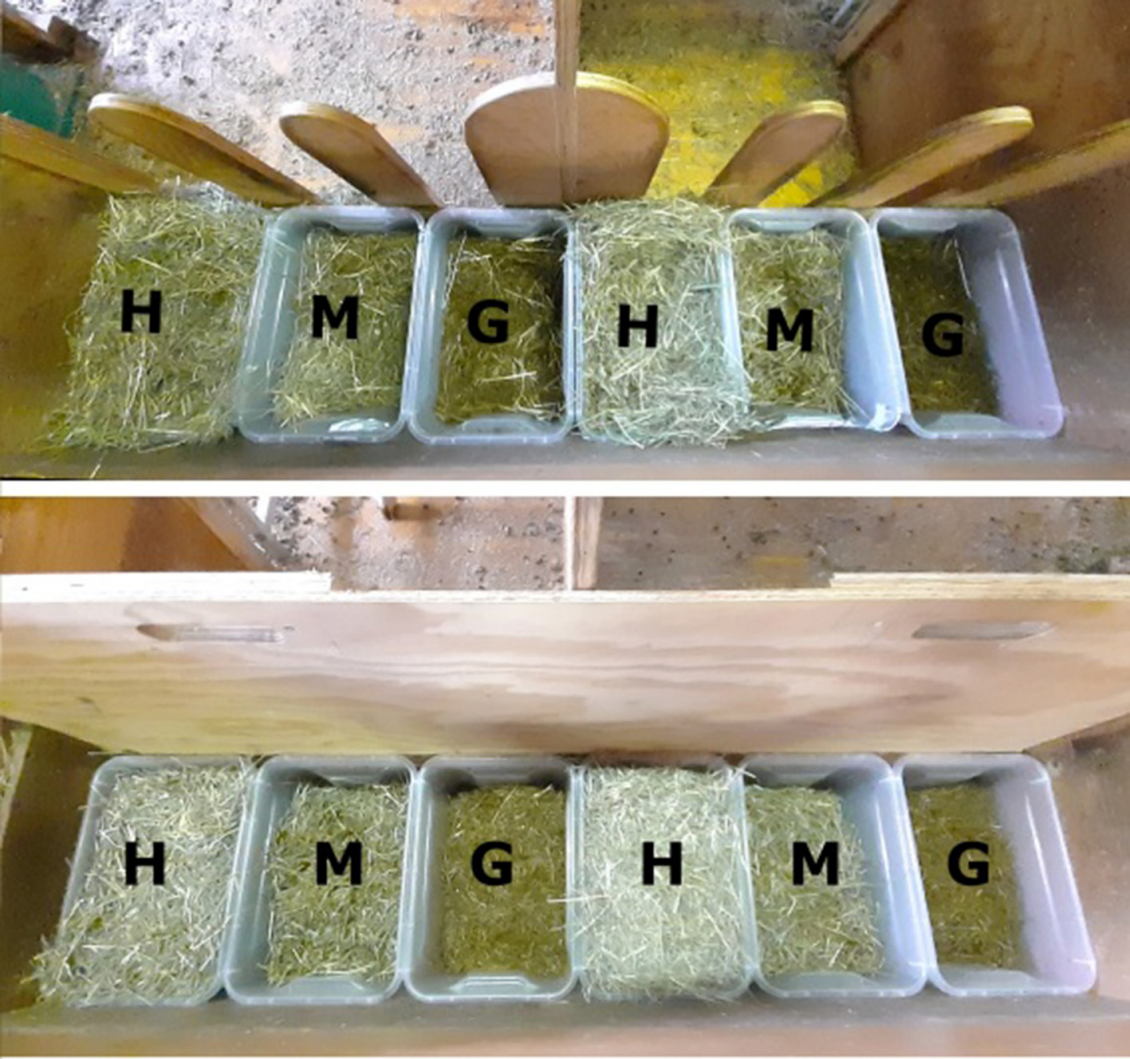
Top view of the feeding trough in the experimental pen (for sheep or goat pairs) with three plastic feed containers per feeding place filled with either grass silage (G), hay (H), or the hay-grass silage mixed ration (M). **(Top)** the animals have access to the feed. **(Bottom)** access to the feed is blocked while the containers are weighed and to measure the animals' first choice after access is given.

The experiments were conducted in a separate outdoor climate stable consisting of four sheep and four goat pens, each large enough to house two animals (mean daily temperature: 9.4°C, BAFU/EUA, MeteoSchweiz). Each pen was 2.4 m × 3.5 m and included an elevated feeding area with two places equipped with a trough. Two pens shared one drinker with *ad libitum* access to water. The two feeding places were separated by a solid wood wall (1.4 m × 0.95 m) to minimize agonistic interactions (23), but allowed visual contact in the area above the trough (Figure 1). The litter area was bedded with sawdust. The goat pens were additionally equipped with round wooden tables for climbing and elevated resting (diameter: 1.1 m; height: 0.8 m).

### Feeds and habituation

Three feeds were used in the experiments. Two of these were single component feeds: chopped hay (H) and chopped grass silage (G) with cutting lengths of ~3–4 cm. The third feed was a mixed ration (M) consisting of the same H and G mixed in a 50:50 dry matter (DM) ratio. G and H had similar protein, fiber, and calculated energy content, as well as a similar botanical composition and were both harvested at the beginning of the flowering stage (Table 1). Both met the nutritional needs of non-lactating sheep and goats (25). To make M, grass silage was added to the mixer wagon (Jaylor Model 5100 Self Propelled, Canada) first. Hay was then added, and the two were mixed for ~10 min. The mixer wagon did not include knives to avoid structural changes on the feeds. M was freshly prepared every day.

**Table 1:** Chemical and botanical composition of grass silage (G), hay (H), and the mixed ration (M).

	**Unit**	**G**	**H**	**M**	**Need goat^1^**	**Need sheep^2^**
Dry matter (DM)	% of fresh weight	31.4	91.3	50.7	–	–
Organic matter	g/kg DM	901	915	907	–	–
Crude protein	g/kg DM	121	108	118	91	81
ADF	g/kg DM	254	263	253	>200^#^	>200^#^
NDF	g/kg DM	428	506	478	>410^#^	>410^#^
NEL^*^	MJ/kg DM	5.5	5.3	5.4	5.1	4.5
APDE^*^	g/kg DM	69	81	76	36	32
APDN^*^	g/kg DM	76	68	75		
Ryegrass	%	75	80–90			
Clover	%	20	10–20			
Herbs	%	5	<5			

ADF, acid detergent fiber; NDF, Neutral detergent fiber; NEL, Net energy for lactation; APDE, Absorbable protein at the duodenum limited by rumen-fermentable energy; APDN, Absorbable protein at the duodenum limited by rumen-fermentable nitrogen.

1 goat with a mean weight of 67.7 kg and mean daily DM intake of 1.5 kg;

2 sheep with a mean weight of 78.1 kg and mean daily DM intake of 1.9 kg;

* calculated according to Agroscope (2021);

# recommended 20% of daily DM intake ADF and 41% NDF (24) (see comments in table).

The animals were familiar with the three feeds from previous experiments (between experiments, animals received uncut hay *ad libitum*). Nevertheless, a habituation phase was conducted prior to the start of the experiment to avoid any neophobic reaction to the feeds (26). Ten days before the experimental phase began, all animals received one of the three experimental feeds *ad libitum* in their group stable; the three feeds were switched daily. In total, the first experimental group (see paragraph Test procedure) received G on 3 days, H on 3 days, and M on 4 days. The second and third group received these feeds for twice and three times as many days as the first group, respectively.

#### Test procedure

The experiment lasted five replicate days for each experimental group. The animals were tested in pairs as stress due to isolation can inhibit feed intake (27). The eight experimental pens were used simultaneously, and the animals were divided into three experimental groups, each group including four pairs of goats and four pairs of sheep.

During the experiment, 50% of the daily ration was offered at 09:00 and 50% at 15:00 via topping up (Table 2). Each animal was offered the three feeds simultaneously in separate plastic containers (28 × 34 × 22 cm). These containers were placed next to each other inside the feeding trough (Figure 1). The positions of the three containers were switched daily in a semi-random order to avoid confounding due to a possible side preference. Each of the three feeds was offered at 100% of the expected daily DM intake, which was estimated using the maximal daily DM intake of similar feeds by the same animals during previous experiments. The overall offer therefore comprised 300% of the animals' anticipated intake.

**Table 2:** Observed dry matter (DM) quantities of grass silage (G), hay (H), and the mixed ration (M) offered and consumed per day and animal.

	**Unit**	**G**	**H**	**M**	**Total**
**Goats**					
DM offered	kg	1.2	1.4	1.0	3.6
Mean DM intake (±SD)	kg	0.9 ± 0.3	0.5 ± 0.2	0.1 ± 0.1	1.5 ± 0.3
Corrected	g/kg LW ^0.75^	36.0 ± 12.1	23.1 ± 6.5	5.0 ±.7	64.1 ± 11.9
Refusals	%	39	55	90	59
Proportional intake	%	57	36	7	100
**Sheep**					
DM offered	kg	1.4	1.9	1.2	4.5
Mean DM intake (±SD)	kg	1.1 ± 0.2	0.5 ± 0.2	0.3 ± 0.2	1.9 ± 0.3
Corrected	g/kg LW ^0.75^	40.9 ± 8.5	18.1 ± 7.5	11.1 ± 6.9	70.1 ± 7.9
Refusals	%	43	66	77	59
Proportional intake	%	59	26	15	100

#### Feed preference recordings

The animals' intake of the three feeds and first feed choices were recorded. Intake was recorded for the animal pairs, and first choice was recorded for each individual. The feed containers were weighed seven times a day at 09:00, 10:00, 12:00, 15:00, 16:00, 18:00, and ~08:30 the following day. Intake of each feed was then calculated for the following time periods: 09:00 to 10:00, 10:00 to 12:00, 12:00 to 15:00, 15:00 to 16:00, 16:00 to 18:00, and 18:00 to 8:30 am the following day. In the following sections, the periods 09:00 to 10:00 and 15:00 to 16:00 are referred to as the “main meals.” These periods correspond to the first hour after feeding, where most feed is consumed per unit of time (28). The time periods from 10:00 to 12:00 and from 16:00 to 18:00, referred to as the “second periods,” are used to compare to main meal results. Feed intake is expressed as grams of DM per kg metabolic life weight (g DM/kg LW^0.75^). To approximate intake per individual, the fresh matter intake recorded per pair was converted to its DM equivalent and divided by the sum of the pair's LW^0.75^.

The animals' first choices of feed were recorded at each time point of weighing the containers as follows. While the containers were weighed, the animals' access to the trough was blocked with a wooden barrier (Figure 1). After the containers were placed back in the trough, the barrier was removed. The first choice was recorded as the feed that was ingested first after the barrier was removed. Individuals that did consume one of the feeds within 1 min after the barrier was removed were recorded as “participating” in the first choice test. Accordingly, individuals that did not do so were recorded as “not participating.”

### Feed analyses

Samples of the fresh M were taken daily, and samples of the H and G were taken on days 1, 3, and 5 of the experimental phase. Samples were dried at 60°C for 48 h to calculate the dry matter content as percentage of fresh matter. For the subsequent chemical analyses, dried samples were pooled per experimental group and ground to pass a 1-mm screen (Brabender rotary mill; Brabender GmbH & Co. KG, Duisburg, Germany). Feed samples were analyzed for exact dry mass content by heating at 105°C for 3 h (prepASH, Precisa Gravimetrics AG, Dietikon, Switzerland) and then incinerating at 550°C until a stable mass was reached to determine the ash content according to ISO 5984_2002. Organic matter was calculated by subtracting the ash content from the dry matter content. The Neutral detergent fiber (αNDF; ISO 16472:2006) and acid detergent fiber (ADF; ISO 13906:2008) contents were analyzed with a fiber analyzer (Fibretherm Gerhardt FT-12, C. Gerhardt GmbH & Co. KG, Königswinter, Germany) and were expressed without residual ash. Neutral detergent fiber (αNDF) was determined after treatment of the sample with heat stable amylase and sodium sulfite and expressed without residual ash after incineration at 600°C for 3 h.

### Statistical analyses

For the statistical analyses and data visualization, we used the open-source software R version 4.2.0 (29). The preference between the three offered feeds was investigated by a log ratio transformation of the feed intake data to avoid the complications otherwise associated with such compositional data (30). A small positive values (0.01 g DM/kg LW^0.75^) was assumed for apparent zero intake to allow the calculation of log ratios (30). The natural log ratios of H and G to M were analyzed using linear mixed-effect models, which was estimated using the *lmer* function of the lme4 R package (31). The model formula in *lme4* syntax is as follows:


$$\begin{array}{l} \text{log}\left(\text{H or G }/\text{ M}\right)\;\sim \;0+\text{Species}:\text{Period}+\left(1\;|\text{ Group}/\text{Pair}\right) \end{array}$$

The model includes an intercept for each period individually for both species as the fixed effect (0 + Species:Period). Furthermore, a random intercept for pair nested within group (1 | Group/Pair) to account for repeated testing of the same animal pair over replicate days and for the potential effects of group affiliation. Only the main meal and second periods were included. The other periods (10:00 to 12:00 and 18:00 to the next day) were excluded as their lengths varied and they included overnight.

**Figure 2:**
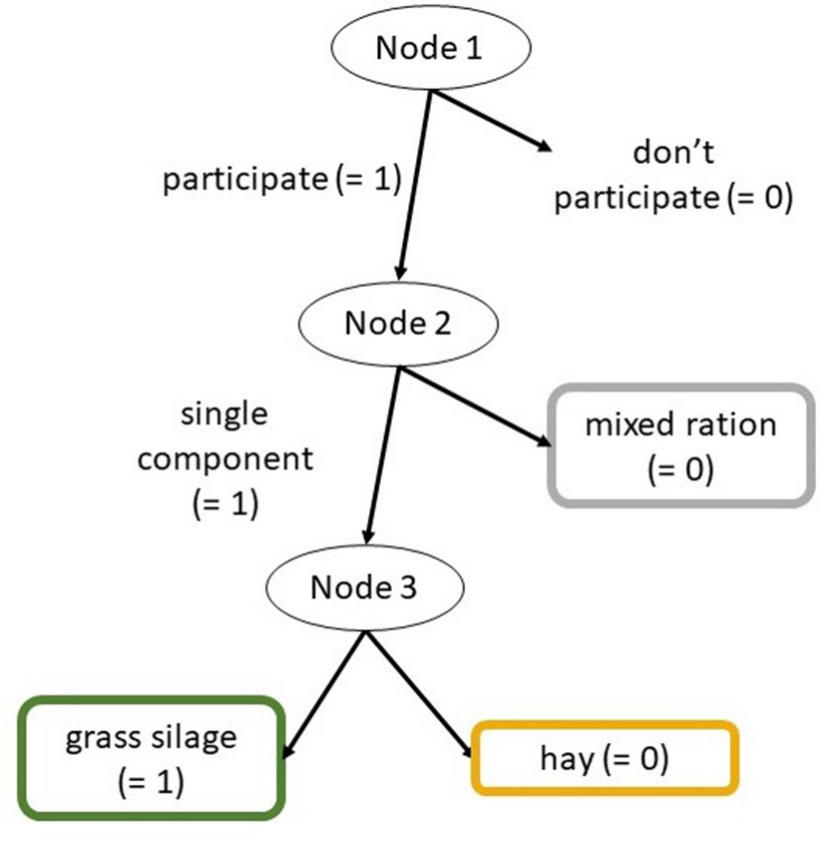
Binary response tree; IRTree model for analyzing first feed choices.

The data on the animals' first choice of feed was analyzed using an item response tree model [IRTree, (32)]. Therefore, the data was encoded as a binary response tree with three nodes (Figure 2). The first node indicated participation (1: yes; 0: no), the second node indicated whether the animal chose a single component or M (1: G or H; 0: M), and the third node indicated whether the animal chose G or H (1: G; 0: H). The IRTree model was estimated as generalized linear mixed model (GLMM) with a binominal response and a logit link function using the *glmer* function from R package lme4. The model formula in *lme4* syntax is as follows:


$$\begin{array}{l} \text{value }\sim \;0+\text{Node}:\text{Species}\\ +\text{Node}:\text{Species}:\text{AmPm}:\text{TimeAfterFeeding}\\ +\left(0\;+\text{ Node }|\text{ Group}/\text{Pair}/\text{Individual}\right)+\left(1\;|\text{ Obs}\right) \end{array}$$

The fixed effects in this model includes an individual intercept for each node for the two species (0 + Node:Species) and an individual slope for the time after feeding for both species separately for the time after the morning and after the afternoon feeding (Node:Species:AmPm:TimeAfterFeeding; the binary variable “AmPm” indicates morning or afternoon). Furthermore, we specified a random intercept for each node for the individual, nested within pair, nested within group (0 + Node | group/pair/individual). This accounts for repeated testing of the same individual and the potential effects of pair and group association. Finally, a random intercept for the observation (1 | Obs) is included to ensure that the binary responses at the three nodes that belong to the same observation are considered to share the same variance. For a detailed discussion of data encoding and model formulation for IRTree GLMMs, see López-Sepulcre et al. (32).

In order to investigate the differences between goats and sheep and between different periods, we tested linear contrasts for the fixed effects in the different models using the *glht* function of the R package multcomp (33). The significance of fixed effects and contrasts were assessed using bootstrapped 95% quantile confidence intervals (CI_95%_), which were determined via parametric bootstrapping as implemented in *bootMer* (10,000 bootstraps, R package lme4). This provides more reliable results than *p*-values based on Wald statistics (31). A significant difference from a null value (typically 0) at the 0.05 level is indicated when the CI_95%_ does not include the null value. Additionally, bootstrapped 95% quantile confidence bands for figures showing population-level fit, as is described by the fixed effects, were obtained using the *predict.MerMod* function (parameter re.form = ~ 0; lme4 package) and the *bootMer* function for parametric bootstrapping (10,000 bootstraps). To overcome the prohibitive computational burden, the IRTree GLMM were refitted to bootstrap samples using the parameter nAGQ = 0 (a faster but less precise method of parameter estimation).

## Results

### Feed intake

**Figure 3:**
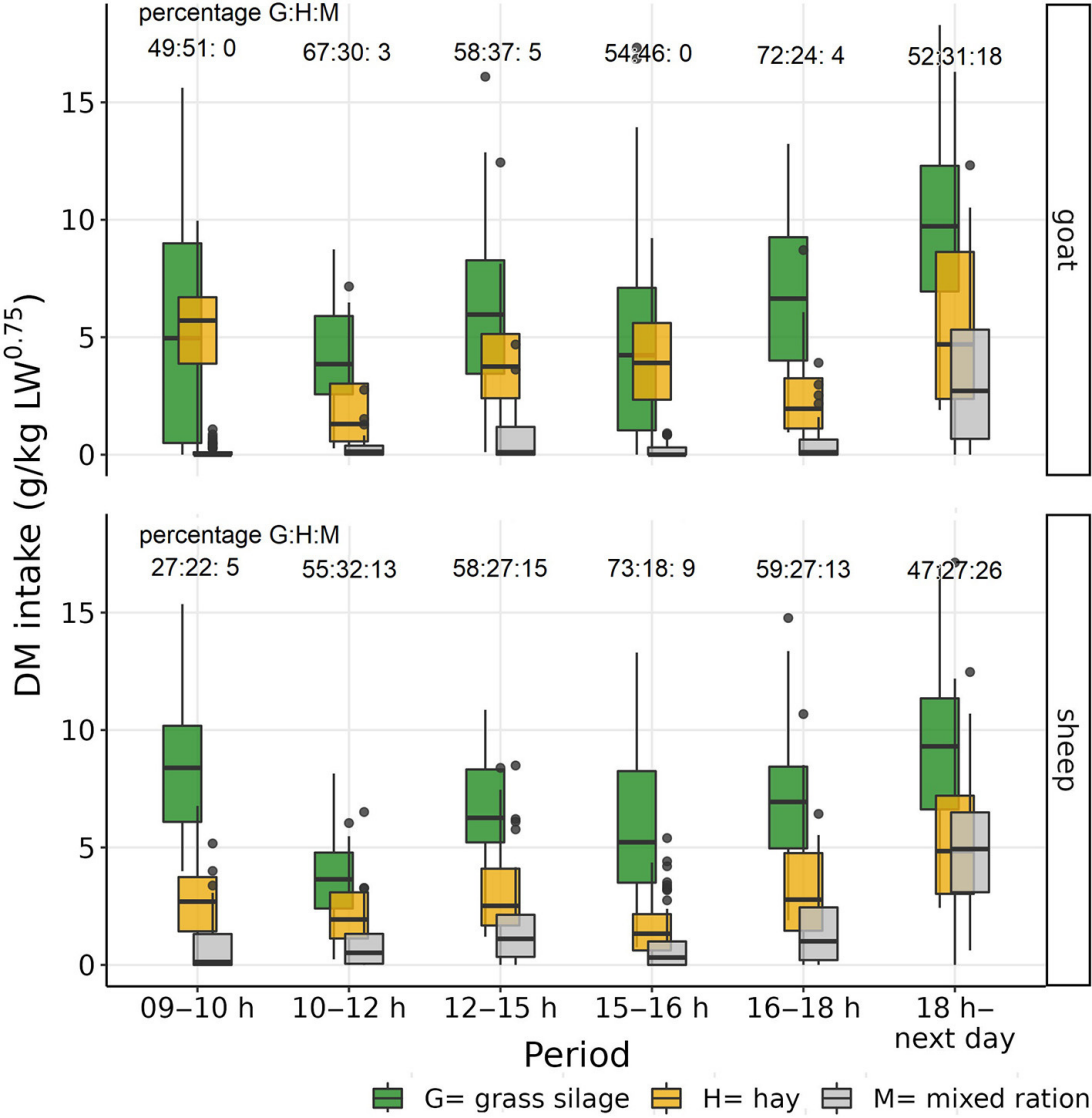
Boxplot of observed individual goats' and sheep's dry matter (DM) intake of the three experimental feeds, grass silage (G), hay (H), and the mixed ration (M), during the different observation periods.

The total mean daily intake (± standard deviation) per individual for goats and sheep was 64.1 ±11.9 g DM/kg LW^0.75^ and 70.1 ± 7.9 g DM/kg LW^0.75^, respectively. The observed mean daily DM intake of M per individual was 5.0 ± 3.7 g DM/kg LW^0.75^ for goats and11.1 ± 6.9 g DM/kg LW^0.75^ for sheep. The proportion of M in the DM intake varied from 0 to 17.7% in goats and from 5.3 to 26.1% in sheep over the different measured time periods. Goats did not eat M at all during the main meals (Table 2; Figure 3).

**Figure 4:**
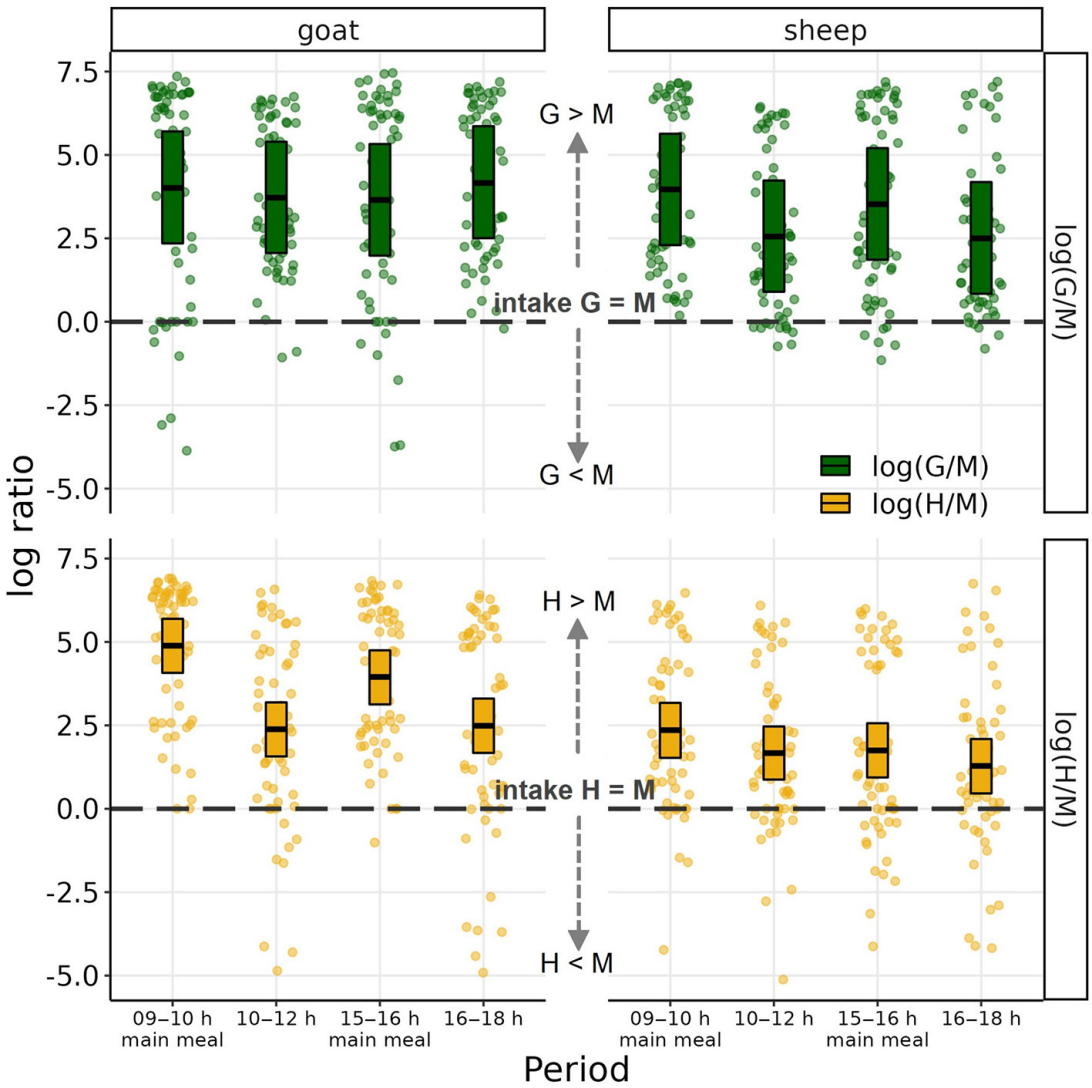
Log ratios of dry matter (DM) intake of grass silage (G) to mixed ration (M) and of hay (H) to M by goats and sheep during the four observation time periods. Boxes represent population-level mean log ratios with bootstrapped 95% confidence intervals as estimated by the linear mixed-effects models. Dots represent observed log ratios.

The results of the mixed effects models for preference as log intake ratios are shown in Figure 4. Log ratios > 0 indicate preference for the single component over the mixed ration, and values < 0 indicate the reverse. Both species ate more G and H than M both overall and during the two main meals and second periods (all CI_95%_ > 0). Comparing the preferences between the species, during the second periods, goats preferred G to M more than sheep (goat-sheep contrast +1.41; CI_95%_ 0.48–2.39) while there was statistically not sufficient evidence for such a between-species difference during the main meals (+0.09; CI_95%_ −0.87 to 1.02). The preference of H to M was higher for goats than sheep; this difference was most apparent during main meals (+2.37; CI_95%_ 1.37–3.33) and less pronounced during the second periods (+0.96; CI_95%_ 0.02–1.98). Comparing the two types of periods within each species, goats showed a higher preference for H to M during main meals than during second periods (main meal-second period contrast +1.98; CI_95%_ 1.39–2.57) while there was statistically not sufficient evidence for such difference in their preference of G to M (−0.11; CI_95%_−0.64 to 0.42). Sheep, on the other hand differed between main meals and second periods in their preference of G to M (+1.22; CI_95%_ 0.68–1.17) and statistically less supported also in their preference for H to M (+0.58; CI_95%_ −0.02 to 1.17; Figure 3).

### First choice

The overall observed rate of participation in the first-choice test (i.e., the animals started eating one of the feeds within the first minute after regaining access to the trough) was 51.3 and 51.9% for sheep and goats, respectively.

**Figure 5:**
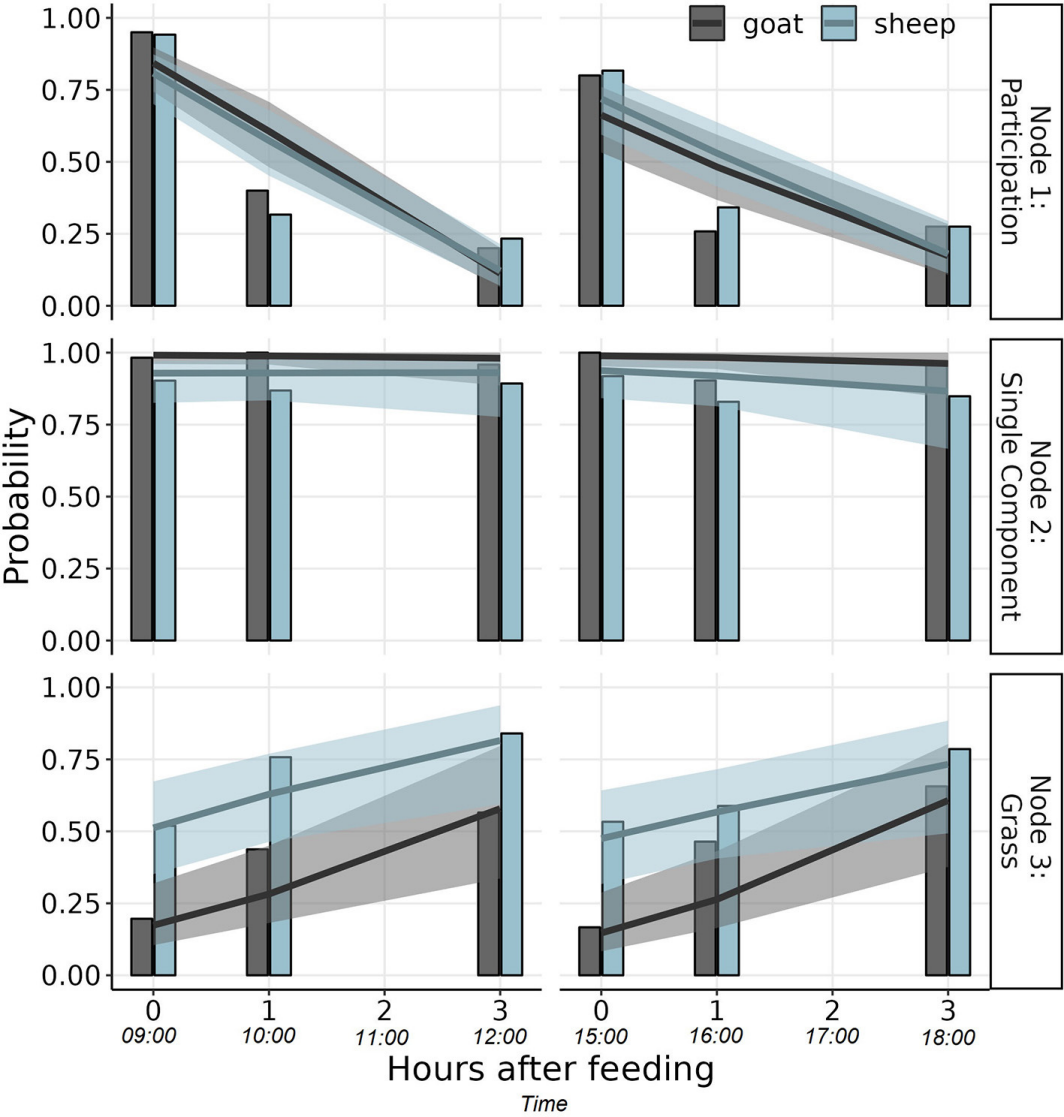
Probability that an animal would choose a feed (i.e., participate) within the first minute after gaining access to the trough (node 1), that the animal would choose a single component (hay or grass silage) over the mixed ration (node 2), and that it would choose grass silage over hay (node 3) at six time points for goats (black) and sheep (blue). Solid lines represent population-level (i.e., described by fixed effects), means with 95% confidence bands as estimated using the IRTree GLMM (shaded area).

The results of the IRTree model are presented in Figure 5. In the morning, the estimated probability of participation (node 1) for both species decreased from over 0.8 at feeding (CI_95%_ 0.74–0.90 for goats; CI_95%_ 0.70–0.87 for sheep) to around 0.5 one hour after feed delivery and to below 0.28 three hours after feed delivery. Similar declines were observed in the afternoon, but initial participation was lower during the afternoon main meal (CI_95%_ 0.53–0.76 for goats; CI_95%_ 0.59–0.81 for sheep) than in the morning. The experimenters observed that three hours after feed delivery (at 12:00 and 18:00), many animals were ruminating.

When the animals participated in the first-choice test, the probability to choose a single component over M (node 2) was estimated over 0.86 throughout the day for both species (all CI_95_ > 0.67; Figure 5), indicating a clear preference (>>0.5) for the single component. This probability was slightly higher for goats than for sheep (goat-sheep contrast on logit-scale: +1.98; CI_95_ 0.71–12.34). No sufficient evidence was found that this choice would differ between morning and afternoon nor over time after feed delivery (Figure 5).

When deciding between the two single components G and H (node 3), goats were more likely to choose H at the initial feeding (09:00, 15:00) as the probability that they would choose G was <0.5 (0.17, CI_95%_ 0.11–0.32 at 09:00 and 0.15, CI_95%_ 0.08–0.29 at 15:00; Figure 5). However, the probability to choose G increased with time after feeding (slope on logit scale: +0.68, CI_95%_ 0.39–0.88) up to that there was no statistical support for a preference between G and H anymore three hours after feeding (CI_95%_ 0.34–0.80 at 12:00; CI_95%_ 0.38–0.80 at 18:00). For sheep, there was no sufficient statistical support for a first choice preference between G and H at any of the sampling time points (all CI_95%_ include 0.5) but similar to goats an increase of the probability to choose G with time after feeding was indicated (slope on logit scale: +0.42, CI_95%_ 0.16–0.68; Figure 5).

## Discussion

This study has investigated whether non-lactating dairy sheep and goats will eat a mixed ration (M) of hay (H) and grass silage (G) when the single components of the mixed ration were offered simultaneously. Although the proportions of feed intake of the three feeds varied throughout the day and differed between the two species, the animals showed a clear preference for G and H and barely consumed M. The same pattern was also seen in the choice of feed consumed first after access to the trough was given.

Prior studies of sheep and goats in indoor feeding conditions have explained feed preferences and selection by nutritional value [goats: concentration ratios and starch types (15), type of protein concentrates (17); sheep: energy density (16); sheep and goats: chemical composition of forages (14)]. In the present study, all three feeds offered had comparable nutritional values and adequately met the nutritional needs of the tested animals (25). Still, in our study, both species refrained from eating the mixed ration, clearly preferring the single-component feeds. Apparently, small ruminants regulate their feed intake based on additional factors not related to the nutritional value of the feed.

A possible explanation for the animals' preference for G and H over M could be the ratio at which the two components were offered in the mixed ration (G:H 50:50). Goats have been shown to select ratios of feed components different from that of a mixed ration calculated to optimally meet their mean nutritional requirements (34). In the present study the nutritional contents of the three feeds did not differ and can therefore not be the reason for the low relative intake of M compared to the single components. The ratio of the two components in M also seem unlikely to be the main reason for the general avoidance by all animals, as at least the goats consumed H and G in a 50:50 DM ratio during their main meals (09:00 and 15:00), which was the same DM ratio of the offered mixed ration. Further studies on different mixing ratios could reveal whether a higher proportion of the one or the other components would increase the acceptance of the mixed ration next to its single components, or whether it is the process of mixing that caused the low relative intake.

The animals might have avoided M because its fixed ratio of the two feeds did not allow for a variation among meals. Görgülü et al. (7) found that free-choice fed goats showed a daytime dependent intake of the different offered feeds and grazing sheep selected different grass species (clover and ryegrass) in the morning than in the afternoon (35). They concluded that ruminants base their feed choice during main meals on attributes that indicate high nutritional value (e.g., rapidly degradable starch). Through post-ingestive feedback (19), they then balance their ruminal milieu during secondary meals by eating different types of feeds, such as those that are high in fiber. Nevertheless, in our study, despite the feeds' comparable nutritional values, the sheep and goats selected different ratios of the feeds throughout the day. Apparently, foraging and eating on a high variety of different plants is a strong behavioral adaption (36, 37) that evolved to ensure a balanced diet (13, 38) and will be performed even if it is not necessary to ensure an adequate supply of nutrients. For example, Scott and Provenza (39) found that lambs diversified their diet by choosing differently flavored rations (apple, anise, fresh forage), even though the rations had similar nutritional values. In another study sheep and goats showed to be sensitive to artificial flavors when choosing feeds as well (21). Since other attributes of the feed than its nutritional contents apparently play a role in sheep's and goats' feed preferences, the present study raises the question of whether mixed rations provide a suitable diet from a welfare perspective, as the mixing itself seems to reduce the palatability.

Our results are consistent with the model developed by Baumont et al. (20) to explain forage intake in small ruminants. This model suggests that the sensory properties of a feed impact the animal's motivation to eat and that the nutritional value of the feed regulates quantity by providing feedback about satiation. Because the feeds we offered had similar nutritional values, sensory properties must be responsible for the animals' feeding behavior in the present experiment. The physical form of feed (e.g., particle size, resistance to fracture, pellets), moisture, smell, and taste have been suggested as the sensory attributes of feeds that impact feed preferences (20). Maybe certain specific sensory attributes of H and G (e.g., dry vs. wet, sour vs. not sour, crispy vs. soft) were substantially diminished or diluted through the process of mixing, resulting in less preferred forms of these attributes. This could explain why the animals avoided M, when they had the choice for H and G but ate normal amounts of M when it was the only feed available (like during the habituation phase). However, only one kind of mixed ration was tested in this study and further investigations are needed to gain a more generalized understanding of small ruminants' acceptance of mixed rations.

The animals' choices of feed consumed first within the 1 min after they regained access to the trough were consistent with their overall feed intake. Both species rarely chose M, and overall intake of M was very low. Sheep's overall intake of G was more than that of goats, and sheep were also more likely than goats to choose G first rather than H. In another study of goats, Abijaoudé et al. (15) found that the feed with the highest daily DM intake was also the preferred one in 3-min choice tests. More recently, Scherer et al. (40) showed that goats' initial feed intake during the first 3 min of a choice-feeding experiment strongly predicts the DM intake over 3 h of feeding. Although it remains unclear which attributes of a feed impact the first choice, the present study confirms that sheep and goats seem to be able to rapidly distinguish the feeds on offer and that their first choice is a good indicator of not only short-term intake (3 h) but also total daily feed intake. Very little research has compared short-term and long-term preferences in ruminants, although Meier et al. (26) mentioned that this distinction could be important in feed choice experiments.

Several aspects that could influence the findings of the present study need further investigation. For instance, all our experimental animals were non-lactating, non-pregnant females of only 2 breeds per species. The external validity of our results are therefore limited. However, a previous study of goats found that feed preference was not impacted by the physiological stage [pregnancy or early or mid-lactation (37)]. Secondly, although the botanical composition and stage of harvest of G and H were similar and the feeds we offered had comparable compositions of macronutrients, their compositions of micronutrients, such as minerals, could have differed. These and other factors could have influenced feed choice between H and G. However, this limitation of the present study does not compromise the main result, that single components were clearly preferred over the mixed ration. Thirdly, on commercial farms, more than two feed components are often used in different mixed rations (41). In order to evaluate whether dairy sheep and goats prefer single-component feeds to mixed rations in general, numerous different mixed rations would have to be tested against their respective feed components. Of particular interest would be the animals' acceptance of total mixed rations, which contain all the components needed to optimally supply the animals' nutritional needs, including minerals and salts as well as concentrates. A consistent clear preference for single components over different mixed rations would indicate that this foraging behavior is a behavioral need of small ruminants. Thus, preventing such behavior would have negative implications for animal welfare.

Our results suggest that sheep and goats prefer the single components of hay and grass silage to a mixed ration of these components. Explanations for this could be that the animals seek variable mixing ratios throughout the day and/or because the sensory stimuli of the single components are lost or significantly reduced through mixing. The present study raises the question of whether mixed-ration feeding is acceptable for sheep and goats from a welfare perspective, as a mixed ration was shown to be less preferred than unmixed single components. Mixed-ration feeding limits small ruminants' natural behavior of selective feeding.

## Data availability statement

The raw data supporting the conclusions of this article will be made available by the authors, without undue reservation.

## Ethics statement

The animal study was reviewed and approved by Swiss Cantonal Veterinary Office Thurgau, Frauenfeld, Switzerland.

## Author contributions

RB: planning and conducting the experiment, data collection, data management, statistical analyses and visualization, and writing. MS: statistical analysis and visualization and co-writing. FD-M: planning and organizing the chemical feed analyses, advising on ruminant nutrition, and co-writing. NK: project management, planning the experiment, and co-writing. All authors contributed to the article and approved the submitted version.

## Funding

The project was funded by the Swiss Federal Food Safety and Veterinary Office (Project number 2.19.e).

## Conflict of interest

The authors declare that the research was conducted in the absence of any commercial or financial relationships that could be construed as a potential conflict of interest.

## Publisher's note

All claims expressed in this article are solely those of the authors and do not necessarily represent those of their affiliated organizations, or those of the publisher, the editors and the reviewers. Any product that may be evaluated in this article, or claim that may be made by its manufacturer, is not guaranteed or endorsed by the publisher.
