# Supplementary material for: Dairy sheep and goats prefer the single components over the mixed ration
Source: Front Vet Sci. 2022 Oct 14;9:1017669. doi: 10.3389/fvets.2022.1017669 (PMC9616466; doi:10.3389/fvets.2022.1017669)
Supplement: Supplementary file 1 [file Data_Sheet_1.pdf]

Supplementary Material

S1. Summary of linear mixed effects model on preferences between grass-silage and mixed ration.

**Formula:**	**log(grass-silage/mixed ration) ~ 0 + Species:Period + (1| Group/Pair)**				
**Random effects:**					
**Groups**		**Name**	**Variance**		**Std.Deviation**
pair:group		(Intercept)	0.94		0.97
group		(Intercept)	1.72		1.31
Residual			4.37		2.09
**Fixed effects:**					
**Effect number**	**Species**	**Period**	**Estimate**	**CI2.5**	**CI97.5**
E1	goat	9-10	4.01	2.34	5.68
E2	goat	10-12	3.72	2.07	5.38
E3	goat	15-16	3.65	1.99	5.35
E4	goat	16-18	4.16	2.50	5.85
E5	sheep	9-10	3.97	2.32	5.67
E6	sheep	10-12	2.55	0.91	4.23
E7	sheep	15-16	3.52	1.85	5.20
E8	sheep	16-18	2.50	0.83	4.18
**Contrasts:**					
**Contrast**		**Hypothesis**	**Estimate**	**CI2.5**	**CI97.5**
Time 1	Goat vs. Sheep	E1 + E3 – E5 – E7 = 0	0.09	-0.87	1.02
Time 2	Goat vs. Sheep	E2 + E4 – E6 – E8 = 0	1.41	0.48	2.39
Goats	Time1 vs. Time2	E1 + E3 – E2 – E4 = 0	-0.11	-0.64	0.42
Sheep	Time1 vs. Time2	E5 + E7 – E6 – E8 = 0	1.22	0.68	1.75

S2. Summary of linear mixed effects model on preferences between hay and mixed ration

**Formula:**	**log(hay/mixed ration) ~ 0 + Species:Period + (1| Group/Pair)**		
**Random effects:**			
**Groups**	**Name**	**Variance**	**Std.Deviation**
pair:group	(Intercept)	0.97	0.98
group	(Intercept)	0.00	0.00
Residual		5.38	2.32
**Fixed effects:**			

Effect number	Species	Period	Estimate	CI2.5	CI97.5
E1	goat	9-10	4.89	4.08	5.71
E2	goat	10-12	2.38	1.56	3.19
E3	goat	15-16	3.95	3.13	4.75
E4	goat	16-18	2.49	1.67	3.28
E5	sheep	9-10	2.36	1.53	3.17
E6	sheep	10-12	1.67	0.85	2.49
E7	sheep	15-16	1.75	0.93	2.57
E8	sheep	16-18	1.28	0.45	2.09
**Contrasts:**					
**Contrast**		**Hypothesis**	**Estimate**	**CI2.5**	**CI97.5**
Time 1	Goat vs. Sheep	E1 + E3 – E5 – E7 = 0	2.37	1.37	3.33
Time 2	Goat vs. Sheep	E2 + E4 – E6 – E8 = 0	0.96	-0.02	1.98
Goats	Time1 vs. Time2	E1 + E3 – E2 – E4 = 0	1.98	1.39	2.57
Sheep	Time1 vs. Time2	E5 + E7 – E6 – E8 = 0	0.58	-0.02	1.17

S3. Summary of Item Response Tree generalized mixed effects model.

**Formula:**	**choice ~ 0 + Node:Species:AmPm + Node:Species:AmPm:TimeAfterFeeding + (0 + Node | Group/Pair/Individual) + (1|Obs)**		
**Random effects:**			
**Groups**	**Name**	**Variance**	**Std.Dev.**
TrialID	(Intercept)	<0.01	<0.01
Name:(Pair:Group)	N1	0.44	0.67
	N2	0.18	0.42
	N3	0.86	0.93
Pair:Group	N1	0.25	0.50
	N2	0.40	0.63
	N3	0.28	0.53
Group	N1	0.01	0.11
	N2	0.24	0.49
	N3	0.05	0.22

Fixed effects:

Effect number	Node	Species	AmPm	TimeAfterFeeding	Estimate	CI2.5	CI97.5
E1	N1	goat	am		0.84	0.74	0.90
E2	N2	goat	am		0.99	0.96	1.00
E3	N3	goat	am		0.17	0.10	0.32

E4	N1	sheep	am		0.81	0.70	0.87
E5	N2	sheep	am		0.93	0.83	0.97
E6	N3	sheep	am		0.51	0.35	0.67
E7	N1	goat	pm		0.66	0.53	0.76
E8	N2	goat	pm		0.99	0.95	1.00
E9	N3	goat	pm		0.15	0.08	0.29
E10	N1	sheep	pm		0.72	0.59	0.81
E11	N2	sheep	pm		0.94	0.84	0.98
E12	N3	sheep	pm		0.47	0.32	0.64
E13	N1	goat	am	slope	0.61	0.48	0.71
E14	N2	goat	am	slope	0.99	0.96	1.00
E15	N3	goat	am	slope	0.28	0.18	0.45
E16	N1	sheep	am	slope	0.57	0.45	0.68
E17	N2	sheep	am	slope	0.93	0.83	0.98
E18	N3	sheep	am	slope	0.63	0.46	0.77
E19	N1	goat	pm	slope	0.48	0.37	0.59
E20	N2	goat	pm	slope	0.98	0.94	1.00
E21	N3	goat	pm	slope	0.26	0.16	0.43
E22	N1	sheep	pm	slope	0.53	0.42	0.64
E23	N2	sheep	pm	slope	0.92	0.81	0.97
E24	N3	sheep	pm	slope	0.57	0.41	0.72

Contrasts:

**Contrast**		**Hypothesis**	**Estimate**	**CI2.5**	**CI97.5**
N1	goat vs sheep	E1 + E7 – E4 – E10 = 0	-0.01	-0.65	0.62
N2	goat vs sheep	E2 + E8 – E5 – E11 = 0	1.97	0.71	12.34
N3	goat vs sheep	E3 + E9 – E6 – E12 = 0	-1.63	-2.30	-0.69
N1	AM vs PM	E1 + E4 – E7 – E10 = 0	0.75	0.37	1.10
N2	AM vs PM	E2 + E5 – E8 – E11 = 0	0.04	-9.82	9.68
N3	AM vs PM	E3 + E6 – E9 – E12 = 0	0.17	-0.28	0.61
N2	AM time slope goat vs sheep	E14 – E17 = 0	-0.28	-7.15	13.78
N2	PM time slope goat vs sheep	E20 – E23 = 0	-0.14	-6.86	3.30
N3	goat time slope	E15 + E21 = 0	0.68	0.39	0.88
N3	sheep time slope	E18 + E24 = 0	0.42	0.16	0.68
